# A systematic review and meta-analysis of the efficacy and safety of arbidol in the treatment of coronavirus disease 2019

**DOI:** 10.1097/MD.0000000000021402

**Published:** 2020-07-24

**Authors:** Xuemei Wang, Ping Xie, Guojuan Sun, Min Zhao, Zhumei Deng, Yunxia Zhou, Shuting Bao

**Affiliations:** a Department of Gynecology, Hospital of Chengdu University of Traditional Chinese Medicine, College of Clinical Medicine, Chengdu University of Traditional Chinese Medicine; b Department of Gynecology, Hospital of Chengdu University of Traditional Chinese Medicine, Chengdu, Sichuan Province, P.R. China.

**Keywords:** 2019 novel coronavirus, arbidol, coronavirus disease 2019, meta-analysis, novel coronavirus pneumonia, severe acute respiratory syndrome coronavirus 2, systematic review, umifenovir

## Abstract

**Background:**

Coronavirus disease 2019 (COVID-19) is highly contagious, and the epidemic has spread to hundreds of countries around the world, and seriously threatens the life safety of people around the world. Arbidol is an antiviral drug with high potential against COVID-19, but evidence of effectiveness and safety is lacking. The systematic review protocol aims to formulate a research plan that can evaluate the efficacy and safety of arbidol for COVID-19.

**Methods:**

The retrieval time will be from the database establishment to June 2020. The retrieval database will include the Cochrane Library, PubMed, Embase, OVID, CNKI, Wanfang, VIP, CBM, etc. The primary outcome will be clinical efficacy, and the secondary results will be accompanying symptoms, time for the temperature to return to normal, time of novel coronavirus nucleic acid turning negative, blood sample test, Computed Tomography examination, length of hospitalization, adverse reactions, and adverse events. RevManV.5.3 software will be used for meta-analysis, and fixed effects model, random-effects model, subgroup analysis, and descriptive analysis will be adopted according to the heterogeneity of the research results.

**Results:**

To provide the latest evidence of clinical efficacy and safety of arbidol in the treatment of COVID-19.

**Conclusion:**

Our study will provide the latest evidence analysis of the efficacy and safety of arbidol for COVID-19, to provide evidence-based medicine for the prevention and control of this epidemic.

**Registration details:**

PROSPERO CRD42020189203.

## 1 Introduction

Coronavirus disease 2019 (COVID-19) is a worldwide infectious disease caused by human infection with severe acute respiratory syndrome coronavirus 2 (SARS-CoV-2).^[1]^ The current epidemic situation has spread worldwide and has become a serious global public health emergency.^[2]^ Mild patients are mainly manifested by fever, cough, and fatigue, and may be accompanied by symptoms such as nasal congestion, runny nose, sputum, dyspnea, pharyngeal pain, muscle pain, diarrhea. Severe patients often present with hypoxemia and dyspnea. With the progress of the disease, crises such as acute respiratory distress syndrome, septic shock, metabolic acidosis that is difficult to correct, coagulation dysfunction, and even life-threatening occur.^[3,4]^

In recent years, the pandemic diseases caused by coronaviruses internationally include COVID-19, and Middle East respiratory syndrome (MERS) in 2012, severe acute respiratory syndrome (SARS) in 2003.^[5]^ Studies have shown that SARS-COV-2 is an RNA virus containing an envelope. SARS-COV-2, SARS-CoV, and MERS-CoV all belong to the β-coronavirus genus.^[6]^ They have high homology in the genome and protein sequence. And there are some similarities in the symptoms caused by viral pneumonia.^[5,7]^

There are currently no antiviral drugs specifically targeting SARS-CoV-2. The clinical medication draws on the experience of SARS, MERS, and other infectious diseases.^[8]^ Conventional treatment methods include antiviral, anti-infective, oxygen therapy, nutritional support, and other symptomatic support treatment.^[9]^ Effectively controlling the progression of the disease, shortening the length of hospital stay, reducing the rate of conversion from mild to severe, reducing serious complications, reducing mortality, and improve the clinical recovery rate are urgent problems to be solved worldwide.^[10]^

Arbidol (Umifenovir) is an antiviral drug with a broad spectrum of activity that is active against many types of viruses, and has been used in many countries for decades.^[11]^ Both laboratory and clinical studies have shown good anti-COVID-19 activity, and its mechanism of action is mainly to activate 2,5-oligoadenylate synthetase to specifically inhibit virus invasion into host cells, thereby blocking viral replication. Besides, the immune system can be regulated by promoting the cells to release interferon and continue to exert antiviral effects.^[12–15]^ In China, clinical guidelines for the diagnosis and treatment of SARS-COV-2 infection recommends the routine use of arbidol for COVID-19.^[16]^

Based on the considerable potential of arbidol to prevent and treat COVID-19, this article intends to systematically evaluate the clinical efficacy and safety of arbidol for COVID-19, and provide a reference for the clinical prevention and treatment of COVID-19 worldwide.

## 2 Research methods

### 2.1 Registration details and ethics

This work has been registered in PROSPERO. The registration number is CRD42020189203. The study is conducted with the requirements of the reporting rules in the “Preferred Reporting Items for Systematic Reviews and Meta-Analyses Protocols (PRISMA-P),” and will strictly comply with the reporting specifications required in the PRISMA statement.^[17]^ This work is a systematic review, the heterogeneity is within the acceptable range, and further meta-analysis will be performed. The data used in this systematic review are all from the published literature, so there is no need to submit it to the ethics committee for review.

### 2.2 Included and excluded criteria

#### 2.2.1 Study design types

All randomized controlled trials (RCT) and quasi-experimental studies of clinically used arbidol for COVID-19 will be included. We will also include studies from the grey literature database, conference literature database, and completed or ongoing trial data from various clinical trial registration databases. Studies <20 participants will not be included.

#### 2.2.2 Participants

Refer to the COVID-19 clinical practice guidelines issued by Infectious Diseases Society of America in the United States,^[18]^ and the Diagnosis and treatment of coronavirus disease-19 (7th trial edition) formulated by China^[16]^ to include patients who meet the COVID-19 diagnostic criteria. Diagnostic criteria for COVID-19 include: throat swab 2019-nCoV nucleic acid positive; confirmed COVID-19; mild type, mild clinical symptoms, no pneumonia manifestations on imaging; normal type, the patient has a fever, respiratory symptoms, imaging findings suggest lung lesions; heavy type, shortness of breath, RR ≥30 times/min, at rest, finger oxygen saturation ≤93%, arterial PaO_2_/FiO_2_ ≤300 mm Hg; crises type, with respiratory failure, shock, and other organ failures. Regardless of the patient's race, nationality, age, education, belief, or economic status.

#### 2.2.3 Interventions

Including clinical trials of arbidol for COVID-19, regardless of the specifications, dosage, manufacturer, frequency, and duration of treatment of arbidol. The experimental group will be arbidol alone, or arbidol combined with other drugs. The control group will be blank, the combination of α-interferon and other antiviral drugs, antibacterial drugs, Chinese patent medicine, Chinese herbal compound, basic treatment, hormone drugs, etc. The control group will not include the clinical treatment of COVID-19 using other methods (aromatherapy, acupuncture, moxibustion, yoga, traditional sports). In this systematic review, the order of the intervention group and the control group in the included clinical studies is not considered. The group containing arbidol is used as the intervention group, and the other drugs or drugs combination is used as the control group. The following types of interventions will be included.

1. arbidol/no treatment.
2. arbidol + α-interferon + other antiviral drugs/α-interferon + other antiviral drugs.
3. arbidol + antibacterial drugs/antibacterial drugs.
4. arbidol + Chinese herbal compound/Chinese herbal compound.
5. arbidol + Chinese patent drug/Chinese patent drug.
6. arbidol + basic treatment/basic treatment.
7. arbidol + glucocorticoid/glucocorticoid.

#### 2.2.4 Outcome measures

##### 2.2.4.1 Primary outcome

The primary outcome will choose clinical efficacy. The proportion of effective patients after treatment was used to evaluate clinical effectiveness.

##### 2.2.4.2 Secondary outcome

1. Accompanying symptoms.
  The change of symptom score after treatment.
2. Time for the temperature to return to normal.
  Days required for the temperature to return to normal after treatment.
3. Time of novel coronavirus nucleic acid turning negative.
  After treatment, the time for the nucleic acid of the novel coronavirus to turn negative.
4. Blood sample test.
  The content of blood sample testing includes white blood cell (WBC), lymphocyte, The proportion of lymphocytes in the blood, C-reactive protein, procalcitonin, etc.
5. Computed Tomography examination.
  Computed Tomography shows the status of lung inflammation absorption after treatment.
6. Length of hospitalization.
  Time from hospitalization to discharge after treatment.
7. Adverse reactions and adverse events.
  Adverse reactions and adverse events recorded in clinical studies.

### 2.3 Literature resources

#### 2.3.1 Literature database search

The electronic retrieval method is adopted, and the retrieval time is from the establishment of a database to June 2020. The retrieval database includes the Cochrane Library, PubMed, Embase, OVID, CNKI, Wanfang, VIP, CBM. The retrieved literature is not restricted by publication status and publication language.

The following in Table 1 lists the search strategies of the PubMed database. The search operators and search fields will be accurately converted to the search strategies of other databases.

**Table 1:**
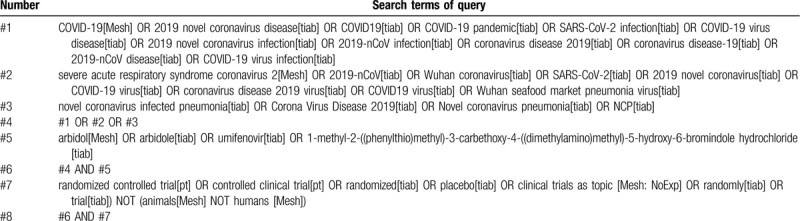
Search strategy for PubMed.

#### 2.3.2 Other literature database

We will continue to retrieve ProQuest, Opengrey, conference literature database ISI proceeding, and various clinical trial registration databases (Clinicaltrials.gov, WHO ICTRP, ISRCTN, Chinese Clinical Trial Register, UK Clinical Trial Gateway, UMIN CTR, UK National Research Register).

### 2.4 Data processing and analysis

#### 2.4.1 Selection of studies

**Figure 1:**
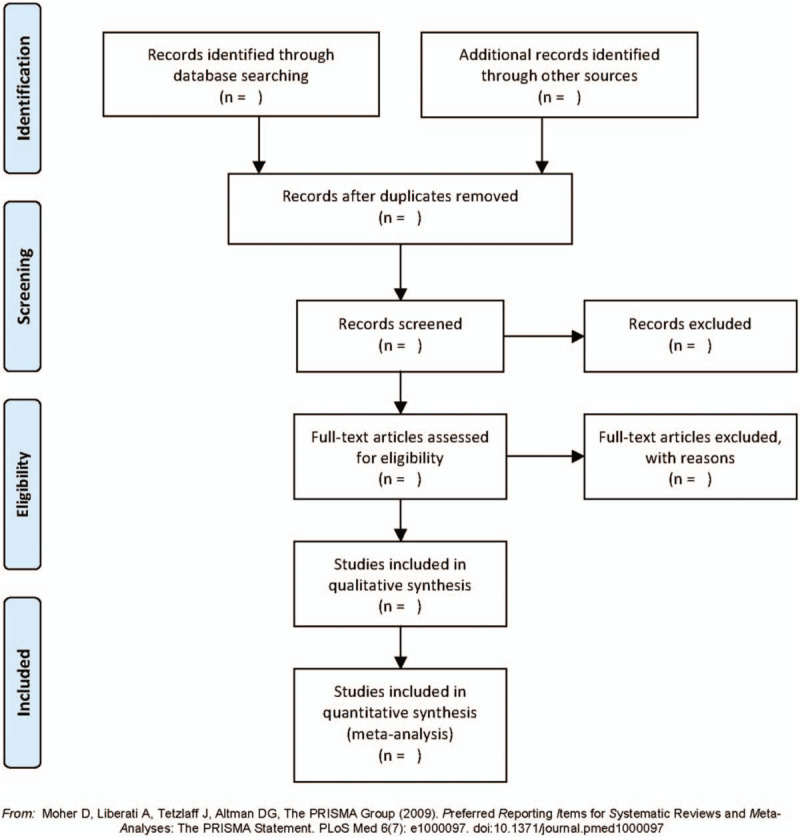
The PRISMA flow diagram of literature screening process. PRISMA = Preferred Reporting Items for Systematic Reviews and Meta-Analyses.

Import the search results into EndNote software V. X9.0 (Thomson Corporation, Connecticut, United States) for document management, grouping, and duplicate check. Titles and abstracts that were reviewed individually by 2 uniformly trained independent reviewers (YZ, SB) based on the inclusion criteria were used to exclude documents that clearly did not meet the inclusion criteria. After the reviewer obtained the full text, each article was evaluated again based on the inclusion criteria. Exclusion descriptions for each article will be recorded in excel. If any differences are encountered, they can be resolved through discussion or referred to a third-party reviewer (XW) to assist in judgment. The research flow chart is shown in Fig. 1.

#### 2.4.2 Data extraction

Two uniformly trained independent reviewers (MZ, ZD) read the full text of all documents that met the inclusion criteria and used pre-made document screening tables to extract the original relevant data. The table consists of 4 parts: general information (document number, title, author, country, publication year, type of funding, journal name, language, ethics, conflict of interest); research method (study design type, study sample size, random allocation method, allocation scheme concealment, blindness method, result data integrity, follow-up period); patient characteristics (age, diagnostic criteria, course of disease); interventions (acupuncture group/control group type, treatment time, frequency of treatment); results (primary and secondary outcomes, outcome measures methods, evaluation criteria, time points, adverse events). The research data records in the literature are missing or unclear, we will try to get in touch with the authors by email.

#### 2.4.3 Assessment of risk of bias

Use the bias risk assessment criteria specified in the Cochrane Review Handbook 5.1.0^[19]^ as the criteria for the quality assessment of the included studies.^[20,21]^ Two trained evaluators independently make bias risk assessments that are included in the literature, check the evaluation results together, discuss and resolve differences, or involve third-party evaluators to assist in judgment. The bias risk assessment includes the following 7 aspects:

(1) the generation of random distribution schemes;
(2) the concealment of distribution schemes;
(3) the use of blind methods for participants and researchers;
(4) the blind method for research results evaluators;
(5) the integrity of outcome data;
(6) selective reporting of research results;
(7) other types of biases.

The results of bias risk assessment in each aspect have 3 levels: high, low, or unclear. If descriptive information on bias risk assessment is missing from the literature, we will try to get in touch with the authors by email for this information.

#### 2.4.4 Measures of treatment effect

The 2-category data were used to calculate the effect index ratio (OR) and 95% confidence interval (CI) to evaluate the effect of arbidol on COVID-19. Continuity variables were calculated by calculating the effect indicator mean difference (MD) or standardized mean difference (SMD) and using 95% CI to estimate the effect of arbidol on COVID-19.

#### 2.4.5 Dealing with missing data

In the case of incomplete research data, we will try to contact the authors by email to supplement the data as complete as possible. All randomly assigned patients, regardless of whether they finally completed the trial or not, will carry out an intention to treat analysis for the test data. For patients who drop out of the trial, the trial data will be carried out with the last observation carry-forward analysis (LOCF) or extreme worst-case analysis (WCA).^[22]^ Finally, a sensitivity analysis was used to determine whether the conclusions of the 2 analyses were consistent.

#### 2.4.6 Assessment of heterogeneity

Chi-square test (*χ*^2^) and *I*^2^ analysis were used to analyze the statistical heterogeneity among the studies. If *P* > .1, *I*^2^ ≤ 50%, heterogeneity is acceptable. If *P* ≤ .1, *I*^2^ > 50%, heterogeneity between studies is considered to be large. Subgroup analysis is conducted for the sources that may lead to heterogeneity. If *P* ≤ .1, *I*^2^ > 75%, meta-analysis will not be carried out, and a qualitative description will be used.

#### 2.4.7 Publication bias

If the number of studies ultimately included is ≥10, the funnel plot is used to test for publication bias, and the Egger test is used to assess the symmetry of the funnel plot. Publication bias, repeated publication bias, selective bias, language bias, and citation bias can all lead to funnel plot asymmetry. We will try to analyze possible causes.

#### 2.4.8 Data analysis and synthesis

RevMan5.3 software (Nordic Cochran Centre, Copenhagen, Denmark) provided by Cochrane Collaboration is used for quantitative synthesis and analysis of data. If *P* > .1, *I*^2^ ≤ 50%, the heterogeneity is accepted, and the fixed effect model is used for analysis; if *P* ≤ .1, *I*^2^ > 50%, the heterogeneity among the studies is large, so the random effect model should be used, but the results should be carefully explained; *P* ≤ .1, *I*^2^ > 75%, the heterogeneity between the studies is too large, cannot be merged, only descriptive analysis.

#### 2.4.9 Subgroup analysis

If there is some heterogeneity among the included studies, if necessary, a subgroup analysis is performed on the factors that may cause the source of heterogeneity. The following is a plan for possible subgroup analysis.

1. Interventions (arbidol alone or in combination with different drugs).
2. The severity of disease (mild type, normal type, heavy type, crises type).
3. Medication timing, dosage, treatment course.
4. Follow-up time (≤3 months, ≤6 months, >6 months).

#### 2.4.10 Sensitivity analysis

Performing a sensitivity analysis can test the stability of the results of the meta-analysis, exclude high-quality studies, low-quality studies, and studies with the largest weights to carry out a meta-analysis, and compare the results of meta-analysis before exclusion. When the sensitivity is high, it is necessary to identify the source of interference factors.

#### 2.4.11 Rating of the quality of evidence

Evaluate the quality of the evidence for all research results with the “Grading of Recommendations Assessment, Development, and Evaluation” software. The quality of the evidence is divided into 4 levels: high, medium, low, and very low. There are 6 kinds of factors that affect the quality of evidence: bias risk, inaccuracy, inconsistency, indirectness, publication bias, and other factors.

## 3 Discussion

The outbreak of novel coronavirus pneumonia in December 2019 broke out and spread rapidly all over the world. It is one of the major epidemic diseases which seriously endangers people's health and public safety. The novel coronavirus is characterized by strong infectious, diverse transmission, and general susceptibility. The latent period of the virus is >7 days. The clinical manifestations are fever, fatigue, nasal congestion, runny nose, dry cough, less sputum, and asthma. Peripheral blood lymphocyte counts are reduced.^[23]^ Imaging examination is most common with multiple ground glass shadows on the periphery of both lungs. The clinical diagnosis depends on epidemiological history and clinical manifestations. A positive 2019-nCoV nucleic acid test in the respiratory tract or blood specimen can confirm the diagnosis.^[24]^ The prognosis of most patients is good, but the prognosis of elderly patients and patients with chronic basic diseases is poor. There are currently no antiviral drugs specifically targeting SARS-CoV-2.^[25]^ The treatment means to refer to the previous diagnosis and treatment experience of treating SARS, HIV, and other viruses, mainly adopt the symptomatic support treatment such as anti-virus, anti-infection, oxygen therapy, antipyretic, nutrition, fluid supplement, etc, to control the progress of the disease and reduce the conversion rate and mortality of serious diseases.^[26,27]^

Arbidol is a synthetic antiviral drug with a broad spectrum of action. It has been used in the prevention and treatment of respiratory diseases caused by a variety of influenza virus infections for decades in many countries.^[28]^ During the SARS epidemic, arbidol was widely used for the prevention and treatment of SARS.^[11,12]^ In China, arbidol is recommended as a COVID-19 treatment drug, with the recommended treatment dose of 0.2 g once, 3 times a day, and the course of medication is mostly 10 days.^[16]^ But in the world, there is no authoritative and unified understanding about the efficacy, timing, dosage, and course of treatment of COVID-19 with arbidol. The arbidol instructions indicated that the incidence of adverse events is about 6.2%, mainly manifested as nausea, diarrhea, dizziness, and elevated serum transaminase.^[29]^ It is not clear whether the adverse reactions of arbidol for COVID-19 are consistent. Therefore, we will conduct a systematic review and meta-analysis to evaluate the efficacy and safety of arbidol for patients with COVID-19.

Inevitably, some limitations exist in this systematic review. Firstly, different manufacturers of arbidol, arbidol treatment dose, and arbidol treatment schedule may have heterogeneity risks. Secondly, it is difficult to obtain complete raw data from the literature, which may cause bias in reporting. We will elaborate on this situation in detail. Thirdly, due to the language environment and database permissions issues, this systematic review only included Chinese and English clinical research literature, which may result in selection bias. It is hoped that this study can provide the latest evidence analysis of the efficacy and safety of arbidol for COVID-19, to provide evidence-based medicine for the prevention and control of this epidemic.

## Author contributions

**Conceptualization:** Xuemei Wang, Ping Xie, Guojuan Sun.

**Data curation:** Xuemei Wang, Min Zhao, Zhumei Deng.

**Formal analysis:** Xuemei Wang, Yunxia Zhou, Shuting Bao.

**Funding acquisition:** Ping Xie, Guojuan Sun.

**Investigation:** Xuemei Wang, Min Zhao, Zhumei Deng.

**Methodology:** Xuemei Wang, Ping Xie, Guojuan Sun.

**Project administration:** Xuemei Wang, Ping Xie, Guojuan Sun.

**Resources:** Xuemei Wang, Min Zhao, Zhumei Deng.

**Software:** Xuemei Wang, Yunxia Zhou, Shuting Bao.

**Supervision:** Xuemei Wang, Ping Xie, Guojuan Sun.

**Validation:** Xuemei Wang, Ping Xie, Guojuan Sun.

**Visualization:** Xuemei Wang, Ping Xie, Guojuan Sun.

**Writing – original draft:** Xuemei Wang.

**Writing – review & editing:** Ping Xie.
